# Electrochemical Detection of Cadmium Using a Bismuth Film Deposited on a Brass Electrode

**DOI:** 10.3390/s25010159

**Published:** 2024-12-30

**Authors:** Milan B. Radovanović, Marija B. Petrović Mihajlović, Ana T. Simonović, Žaklina Tasić, Milan M. Antonijević

**Affiliations:** Technical Faculty in Bor, University of Belgrade, Vojske Jugoslavije 12, P.O. Box 50, 19210 Bor, Serbia; mradovanovic@tfbor.bg.ac.rs (M.B.R.); mpetrovic@tfbor.bg.ac.rs (M.B.P.M.); asimonovic@tfbor.bg.ac.rs (A.T.S.); ztasic@tfbor.bg.ac.rs (Ž.T.)

**Keywords:** bismuth film electrode, brass, cadmium detection, square-wave stripping voltammetry, electrochemical impedance spectroscopy

## Abstract

Cadmium is one of the most dangerous pollutants found in the environment, where it exists mainly due to human activities. High cadmium concentrations can cause serious problems, which is why the detection and determination of Cd is one of the most important tasks. Electroanalytical methods provide rapid and accurate results in the detection of cadmium in various solutions. In this study, the possibility of using a bismuth film electrode deposited on a brass surface and electroanalytical techniques for the detection of cadmium is investigated. The bismuth film was deposited on the surface of the brass electrode using a chronoamperometric technique. Cyclic voltammetry and electrochemical impedance spectroscopy were used to characterize the synthesized bismuth film electrode. The current peaks obtained by anodic square-wave stripping voltammetry under optimized conditions showed a linear relationship in the investigated concentration range of cadmium. The study of the interference of different cations (Cr^3+^, Mn^2+^, Zn^2+^, Ca^2+^, K^+^, Mg^2+^ and Na^+^) showed that the tested cations have no influence on the determination of Cd^2+^ ions in the investigated solution. This finding provides a good opportunity for the use of the synthesized electrode in real samples.

## 1. Introduction

The presence of heavy metal ions in various aqueous solutions causes serious problems in the environment. Urban wastes, mining and metallurgical industries, and agriculture are the main sources of heavy metals in river water and smaller watercourses [1]. Accordingly, various analytical methods for the detection of heavy metal ions have been developed and successfully used in recent decades [2,3,4,5]. These methods usually require large equipment, complex sample preparation, and high cost. On the other hand, electroanalytical methods have many advantages over other analytical techniques, such as low-cost equipment, simpler and faster sample preparation, rapid detection, etc. Among the electroanalytical methods, stripping techniques are very useful because of their short detection times and excellent sensitivity. Stripping techniques are often used to determine very low levels of heavy metals and organic pollutants in various media [6,7,8,9]. Anodic stripping voltammetry is a suitable electroanalytical method for the determination of heavy metals in various samples. The importance and wide applicability of anodic stripping voltammetry stems from its ability to determine metals in very small amounts with acceptable accuracy. Mercury-based electrodes, especially MDPE, have been predominantly used as working electrodes in electroanalytical techniques [10,11,12]. It is also known that mercury is a toxic element, and, accordingly, many researchers have endeavored in recent years to find environmentally friendly alternatives for mercury electrodes [13,14,15,16,17,18,19,20].

Electrodes that play a very important role in the removal of mercury from electroanalytical methods are mercury-free film electrodes [21,22,23]. Bismuth is a readily oxidizable metal used for the fabrication of sensors and the determination of electrochemical processes occurring at negative potentials [24]. Bismuth-modified electrodes are one of the acceptable alternatives since bismuth is an environmentally-friendly element [25,26,27]. Another advantage of bismuth film electrodes is the possibility of the detection of trace metals without prior removal of oxygen from the working solutions. Nowadays, bismuth film electrodes (BiFEs) and bismuth bulk electrodes (BiBEs) are some of the most commonly used modified electrodes [28,29]. In addition to the differences in the construction of the electrodes, they also differ in their properties of use. Bismuth film electrodes were prepared by depositing bismuth on a suitable substrate such as glassy carbon [30,31,32], graphite [33,34] or copper [35]. All these substrates were modified ex situ or in situ with bismuth, and the obtained BiFEs were used for the determination of heavy metals and various organic compounds in different solutions [36,37,38].

The aim of this work is to determine parameters for the preparation of a bismuth film electrode on a brass substrate for the useful detection of cadmium. Determining the optimal parameters for the detection of cadmium was also the focus of this manuscript. In this work, brass was investigated as a substrate for the synthesis of BiFE, since its economy cannot be neglected in the selection of materials. On the other hand, brass is a readily available material that can be recycled, which also contributes to its wide use. Brass has other advantages over the widely studied materials that have been used as substrates for the synthesis of BiFEs. One of the most important is that it is suitable for processing, which enables the easy fabrication of sensors of different shapes and dimensions, which would contribute to a wider application of sensors and microsensors, especially in industrial conditions. In addition, the work observed the nucleation mechanism and growth kinetics of Bi(III) deposits on the brass surface during the BiFE synthesis process. This aspect of the synthesis of sensor electrodes has been insufficiently studied in the literature and no adequate data are available. According to the literature data available to the authors, there is no work on brass as a substrate for the synthesis of BiFE and, in particular, no work dealing with the nucleation mechanism and growth kinetics of Bi(III) deposits.

## 2. Experimental

### 2.1. Bismuth Film Formation

Hydrochloric acid (HCl, ALKALOID, Skopje, North Macedonia), bismuth(III) nitrate pentahydrate (Bi(NO)_3_∙5H_2_O), acetic acid (CH_3_COOH, ASTRAchem, Zemun, Serbia), sodium acetate (CH_3_COONa), and cadmium chloride (CdCl_2_∙2 ½ H_2_O, Kemika, Zagreb, Croatia) were used to prepare the solutions. All reagents used for the studies were of the highest purity.

The bismuth film was formed by deposition on a brass (Cu37Zn) substrate at a constant potential. Electrodeposition of the bismuth film was performed ex situ in 1M HCl solution with the addition of 0.02M Bi(NO)_3_. The brass electrode was polished with Al_2_O_3_ (0.3 μm, Buehler, Lake Bluff, IL, USA) until a mirror-smooth surface was obtained, then it was rinsed with distilled water and air dried. The prepared electrode was immersed in an HCl solution with the addition of Bi^3+^ species, and the bismuth film was deposited by chronoamperometry at different potentials (−0.1 V, −0.12 V, −0.15 V, −0.2 V, and −0.3 V vs. SCE) for 300 s ex situ. Bismuth film formation occurred in HCl solution due to the suppression of the hydrolysis of bismuth. The deposit on the brass surface was observed immediately after removing the electrode from the HCl solution, which was evidence of a successfully formed film.

### 2.2. Instrumentation and Methods

A three-electrode cell was used for the voltammetric measurements. The working electrode was a brass electrode with a bismuth film applied to its surface, a platinum wire served as the counter electrode, and a saturated calomel electrode (SCE) was used as the reference electrode. All electrochemical measurements were performed using a potentiostat (IVIUM XRE, IVIUM Technologies, Eindhoven, The Netherlands) and appropriate software.

Potentiodynamic measurements were performed from −1.4 V to −0.4 V vs. SCE at a scan rate of 10 mV/s, while anodic square-wave stripping voltammetry measurements were performed under the following conditions: Frequency 10 Hz, step potential 5 mV, pulse amplitude 50 mV, deposition potential −1.2 V vs. SCE, and accumulation time 300 s. Potential was cycled from −1.1 V to −0.6 V vs. SCE after an equilibrium time of 15 s.

Electrochemical impedance measurements were performed at different potentials (−1.1, −0.9, and −0.6 V vs. SCE) in the frequency range of from 65 kHz to 0.5 Hz with a single amplitude perturbation of 10 mV.

The surface morphology of the brass electrode before and after the deposition of Bi(III) was examined using a Tescan VEGA 3 LM scanning electron microscope in conjunction with an Oxford EDS X-act Inca 350 system.

All measurements were performed in acetate buffer solution (pH 4.35) without and with the addition of Cd^2+^ in amounts ranging from 9.5 × 10^−7^ M to 1.33 × 10^−5^ M at room temperature. All measurements were repeated at least three times.

Bor Lake samples were taken from Bor Lake, Bor, Serbia, and used as real samples for the determination of Cd^2+^. The real samples were diluted 10-fold in acetate buffer and different concentrations of Cd^2+^ were added. The Cd^2+^ concentrations used for the preparation of the real samples were 5.3 × 10^−6^ M (BL 1), 6.65 × 10^−6^ M (BL 2), and 9.5 × 10^−6^ M (BL 3). After the preparation of the real samples, the square-wave stripping voltammetry measurements were carried out.

## 3. Results and Discussion

### 3.1. Chronoamperometry Measurements

**Figure 1:**
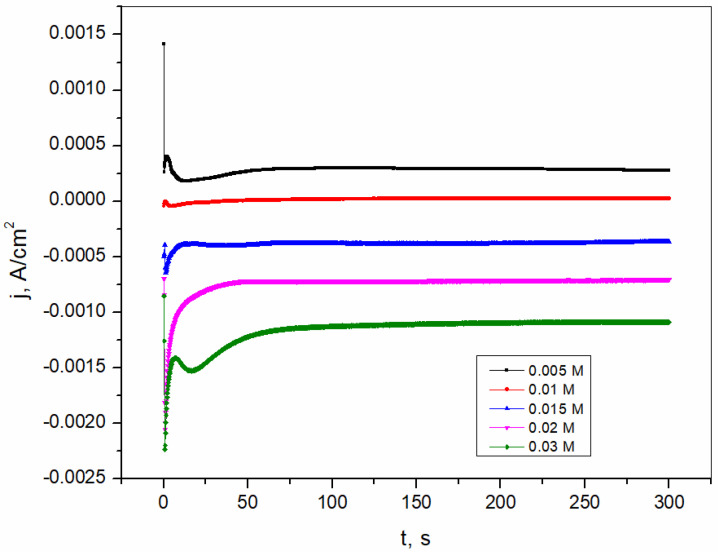
The chronoamperometry curves of the formation of the bismuth film on the brass electrode in the presence of different concentrations of Bi(III).

The chronoamperometry (CA) curves shown in Figure 1 illustrate the process of bismuth layer formation on the brass substrate. Moreover, the CA curves show the influence of Bi(III) concentration on the quality of the formed film under the tested conditions. The obtained curves show that with increasing Bi(III) concentration the current density decreases which reveal that the formed film becomes thicker as well as the surface area covered with the film increases. The formation of bismuth cores during deposition on the substrate surface was accompanied by the formation of a diffusion zone around the formed cores. The current peak observed on the CA curves is the result of the nucleation process. Thereafter, the current is limited by the diffusion of Bi(III) ions to the electrode surface. The chronoamperometry curve shows that the current reaches a plateau in a long time interval, suggesting that the reaction is kinetically controlled by the mass transport of Bi(III) ions to the electrode surface [39].

**Figure 2:**
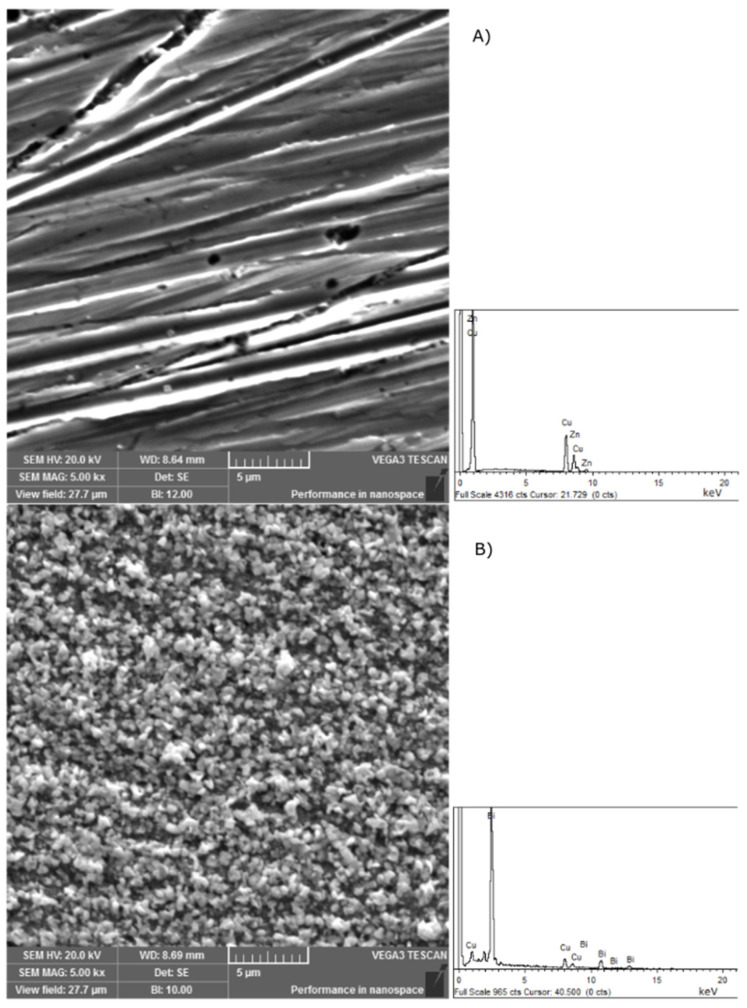
Scanning electron microscopic image of (**A**) bare brass electrode and (**B**) Bi(III) film deposited on the brass electrode.

Figure 2 shows the surface of a freshly polished brass surface and the surface of a bare brass electrode on which bismuth(III) has been deposited. The figure shows the clear difference in the morphological state of these two surfaces. The polished brass surface is smooth and shows visible traces of polishing. The SEM results show that a bismuth film has formed after chronoamperometric treatment at a potential of −0.15 V vs. SCE for 300 s in HCl solution with the addition of 0.02 M Bi^3+^. The EDS results also confirm this conclusion by detecting bismuth on the surface of the brass electrode.

### 3.2. Nucleation Modeling

**Figure 3:**
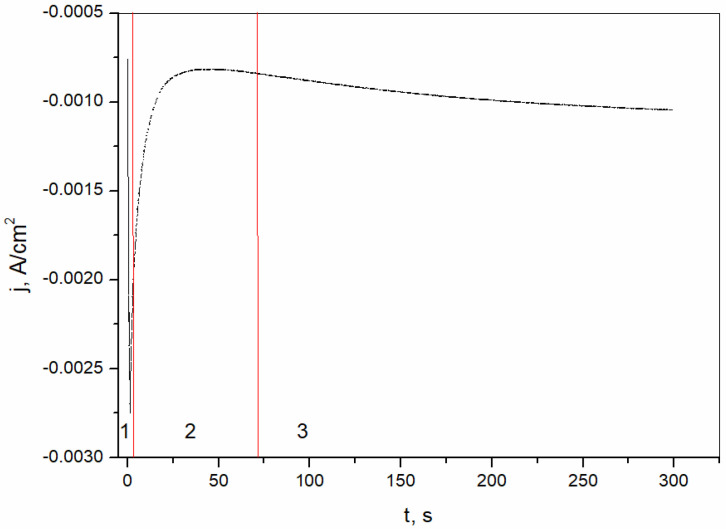
Bi(III) deposition on the brass substrate at −0.15 V vs. SCE. 1—charge of the double layer; 2—formation and growth of the nuclei; 3—growth of the formed bismuth film.

The nucleation mechanism and growth kinetics of the Bi(III) deposition process on a brass substrate were investigated. Figure 3 shows the Bi(III) deposition on the brass substrate at a potential of −0.15 V vs. SCE in a solution with an amount of 0.02 M Bi^3+^.

**Figure 4:**
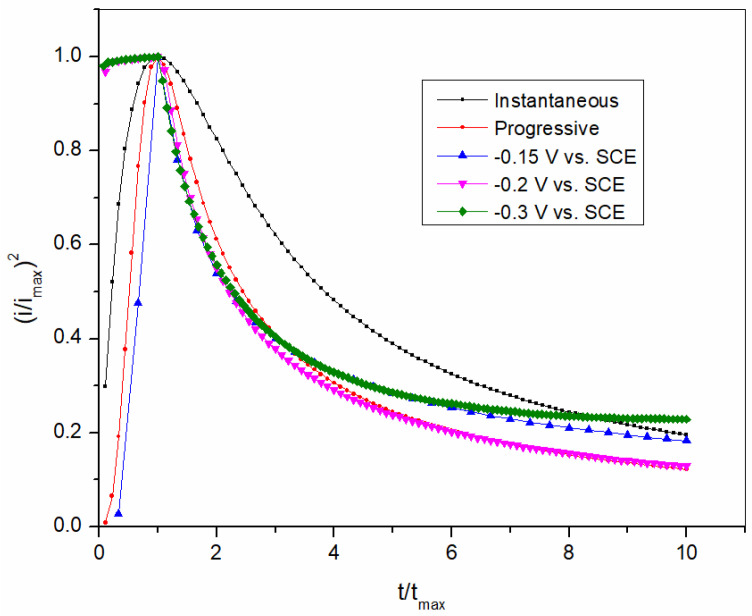
Experimental plots of (*i*/*i_max_*)^2^ as a function of *t*/*t_max_* compared with theoretical curves for instantaneous and progressive nucleation.

The curve shows the characteristic regions, the first of which corresponds to the charge of the double layer. The second region is related to the nucleation and growth of the nuclei. The last region is associated with the growth of the formed bismuth film on the electrode surface. In the last region, a decrease in current density is observed, which is due to the decrease in the concentration of electroactive species on the electrode surface [40]. In order to further investigate the kinetic parameters of the deposition process of the bismuth film on the brass surface, an evaluation of the nucleation process was performed, and the results are shown in Figure 4. The experimental data were estimated in accordance with the theoretical calculation using the model derived by Scharifker and Hills (SH) [41]. The SH model of instantaneous and progressive nucleation is shown in the following equations [39]:

(1) $${\left(\frac{i}{i_{max}}\right)}^{2}=1.9542\left(\frac{t_{max}}{t}\right){\left[1-exp\left(-1.2564\left(\frac{t}{t_{max}}\right)\right)\right]}^{2}$$

(2) $${\left(\frac{i}{i_{max}}\right)}^{2}=1.2254\left(\frac{t_{max}}{t}\right){\left[1-exp\left(-2.3367{\left(\frac{t}{t_{max}}\right)}^{2}\right)\right]}^{2}$$

Here *i* and *i_max_* stand for current density and maximum current density, respectively; t is the time and *t_max_* is the time when the current reaches an i_max_ value.

When the nucleation rate is high, nucleation occurs immediately. According to this mechanism, all active sites on the electrode surface are quickly covered, and then the growth of the nuclei follows. If the nucleation rate is low, progressive nucleation takes place. In this case, the nuclei form continuously during an overpotential period [42]. The curves shown in Figure 4 represent the experimentally and theoretically obtained results in a dimensionless form, which allows and facilitates the representation of the instantaneous and the progressive nucleation. The curves obtained show that progressive nucleation is a model that fits the Bi(III) deposition process at different potentials.

The nucleation rate of Bi(III) on the electrode (*AN*_∞_) and the density of saturated Bi(III) nucleation number (*N_s_*) as parameters of SH models of progressive nucleation were calculated according to the equations [43]:

(3) $${AN}_{\infty }=\frac{4.6733}{t_{max}^{2}\pi k^{\prime }D}$$

(4) $$N_{s}={\left(\frac{{AN}_{\infty }}{2k^{\prime }D}\right)}^{\frac{1}{2}}$$

(5) $$k^{\prime }=\frac{4}{3}{\left(\frac{8\pi CM}{\rho }\right)}^{\frac{1}{2}}$$

where *A* stands for the area of the electrode, *D* is diffusion coefficient, *C* is the Bi(III) concentration, *M* and *ρ* represent the molar mass and density of bismuth, respectively.

The results presented in the Table 1 show that *AN*_∞_ and *N* decrease with the negative shift of the potential, indicating that the growth rate of the nuclei is highest at a potential of −0.15 V vs. SCE.

### 3.3. Electrochemical Characterization of the Bismuth Film on Brass Substrate

#### 3.3.1. Electrochemical Window for the BiFE

The bismuth film was formed on the Cu37Zn electrode according to the procedure described in Section 2.1. The bismuth film, obtained by chronoamperometry at a potential of −0.3 V vs. SCE, achieved low adhesion to the brass substrate as the film was removed by the water jet. Bismuth films formed at other potentials showed better adhesion to the substrate and reasonable mechanical stability.

After the deposit was formed, the bismuth film electrode was immersed in an acetate buffer solution, and CV measurements were performed to determine a potential window limit for the appropriate use of the electrode in the detection and quantitative determination of heavy metals. Theoretical considerations reveal that bismuth film electrodes have been used for measurements of electrodeposited elements with standard potentials more negative than bismuth, which is one of the main limits for the use of BiFEs [25,44,45].

**Figure 5:**
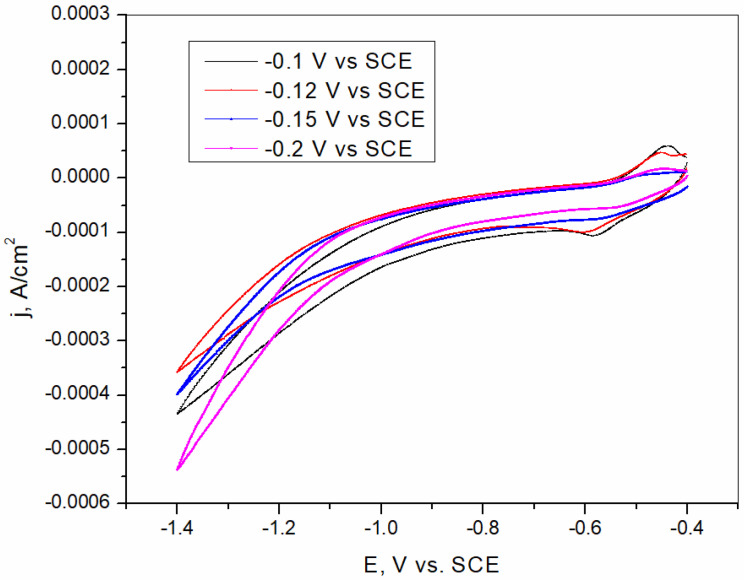
Cyclic voltammograms of bismuth film electrode formed ex situ at different potentials in 0.1 M acetate buffer. Sweep rate 10 mV/s.

BiFE shows a negative potential window, which can be wider compared to the carbon film electrode [46]. The width of the potential window depends strongly on the electrode substrate and the composition of the solution in which the electrode was immersed [47]. The CV curves presented in Figure 5 show the negative limit of the potential window of BiFE formed at different potentials in acetate buffer solution. In addition to the limit of the potential window, the CV curves also show the intensity of the background currents, which is low despite the measurements being performed in a naturally aerated solution, indicating a low susceptibility of the bismuth film electrode to background reactions [44,48,49]. Bismuth film electrodes usually have a high hydrogen overpotential, which allows for the detection of heavy metals in a wider range of cathodic potential [50,51]. According to the CV curves, the high hydrogen overpotential of the bismuth film electrodes tested allows for the detection of cadmium without interference from the reduction of hydrogen. The curves shown in Figure 5 indicate that the electrochemical behavior of electrodes obtained at different potentials and recorded in an acetate buffer solution is similar.

CV curves of bismuth film electrodes formed by chronoamperometry at −0.1 V vs. SCE and −0.12 V vs. SCE recorded in acetate buffer solution demonstrate the presence of oxidation and reduction peaks at potentials of −0.4 V vs. SCE and −0.6 V vs. SCE, respectively, due to the reduction and oxidation of Bi species [52,53]. This limits the application of bismuth film electrodes formed at these potentials.

No current peaks are visible on other CV curves obtained after the formation of the bismuth film by chronoamperometry at a more negative potential, indicating that the properties of the bismuth film depend on the potential used for its formation. The CV curve obtained after the formation of the bismuth film at a potential of −0.15 V vs. SCE shows the best combination of hydrogen overvoltage, background currents, and width of the potential boundary. Therefore, the potential of −0.15 V vs. SCE was determined to be the most suitable for the deposition of the bismuth film on the brass surface and was used to prepare the bismuth film electrode for further measurements. The bismuth film formed at a potential of −0.15 V vs. SCE was more continuous and dense compared to the deposited bismuth films at potentials of −0.1 V vs. SCE and −0.12 V vs. SCE. The formation of the denser film was caused by the different packing of bismuth particles as well as higher growth rates [54]. On the other hand, at potentials of −0.2 V vs. SCE and −0.3 V vs. SCE, the formed film became darker, and the surface was rougher due to the formation of dendrites. This is due to the influence of the hydrogen reduction reaction in bismuth film formation at these potentials. The dendrites adhere weakly to the electrode surface, leading to effortless detachment of the dendrites from the surface. This leads to instability of the formed film, which is one of the main reasons why bismuth deposits formed at −0.2 V vs. SCE and −0.3 V vs. SCE were not suitable for further applications [54,55].

**Figure 6:**
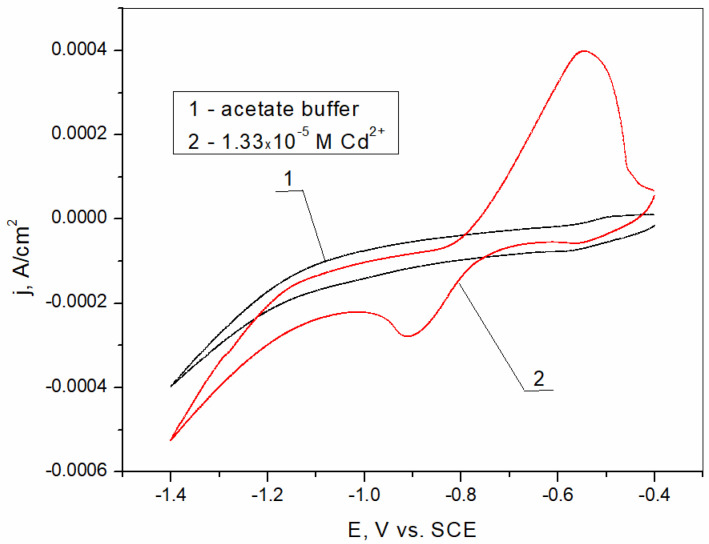
Cyclic voltammogram of bismuth film electrode formed ex situ in HCl solution at a potential of −0.15 V (1) in bare acetate buffer solution; (2) in acetate buffer solution with addition of 1.33 × 10^−5^ M Cd^2+^.

The CV curve obtained in the acetate buffer solution with the addition of cadmium is shown in Figure 6. The bismuth film electrode used for the measurements was prepared chronoamperometrically at a potential of −0.15 V vs. SCE, according to the previous discussion. The results obtained show the existence of reduction and oxidation peaks at a potential of −0.91 V vs. SCE and −0.55 V vs. SCE, respectively. The current peaks suggest charge transfer processes represented by the following equations [56]:

(6) $$\mathrm{Reduction}:\;{Cd}^{2+}+2e^{-}={Cd}^{0}$$

(7) $$\mathrm{Oxidation}:\;{Cd}^{0}-{2e}^{-}={Cd}^{2+}$$

**Figure 7:**
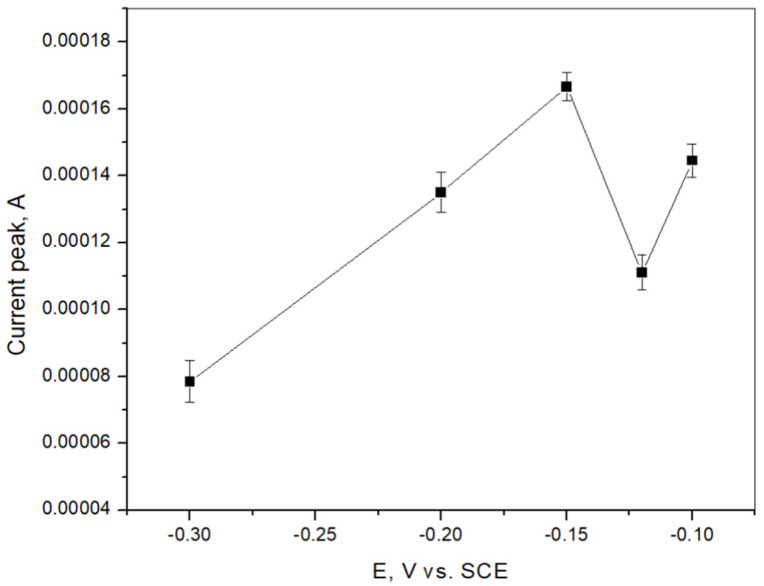
Effect of potential used for formation of BiFE on the stripping signal for cadmium determination in acetate buffer solution. C_Cd_^2+^ = 1.33 × 10^−5^ M. Mean values of three measurements are presented with corresponding error bars.

Figure 7 shows the influence of cadmium stripping signals obtained in the acetate buffer solution at BiFE formation potentials ranging from −0.3 V vs. SCE to −0.1 V vs. SCE. The current peak was strongest when BiFE was produced at a potential of −0.15 V vs. SCE, indicating that this potential is optimal for bismuth film electrode production. The highest stripping signal is the result of the less dense packing of the bismuth particles at a deposition potential of −0.15 V vs. SCE compared to the dense packing of the Bi particles at deposition potentials of −0.1 V vs. SCE and −0.12 V vs. SCE. At potentials of −0.2 V vs. SCE and −0.3 V vs. SCE, the hydrogen reduction reaction affects the deposition of bismuth, resulting in a lower stripping signal [54].

#### 3.3.2. Electrochemical Impedance Spectroscopy Measurements

**Figure 8:**
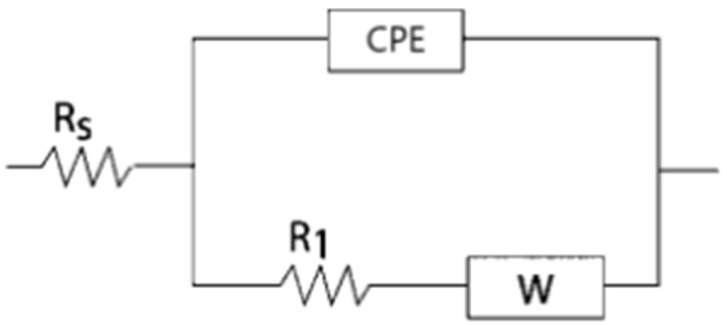
Electrical equivalent circuit for BiFE in acetate buffer solution with and without the addition of Cd^2+^.

Bismuth film electrodes were characterized by electrochemical impedance spectroscopy to understand how different conditions of bismuth film formation affect the structure, morphology, and, ultimately, the successful application of BiFE in anodic stripping voltammetry techniques. The equivalent electrical circuit used to fit the obtained EIS curves is shown in Figure 8. In all presented impedance plots, the symbols represent the data obtained during the experimental measurements, and the solid lines were obtained by fitting with the equivalent circuit.

The presented model is used to describe the electroanalytical process when the rate-determining step is a combination of kinetic and diffusion-controlled processes [21]. *R_S_* in the equivalent circuit represents the cell resistance, and *R*_1_ denotes the polarization resistance, which corresponds to the charge transfer resistance in the assumed equivalent circuit and is a parameter indicating the extent to which the formed film resists the transfer of electrons to electroactive species in solution. A lower polarization resistance means that electron flow is easier, resulting in faster oxidation and a stronger stripping signal [21,57]. CPE is a constant phase element represented by *Q* and *n*. *Q* indicates the non-ideal capacitance and has no physical meaning. It is only used for matching. CPE was used as a substitute for capacitors in impedance spectrum analysis because the impedance loop obtained in experiments is not an ideal semicircle due to the roughness and inhomogeneity of the electrode surface. The parameter *n* was used as an indicator of the roughness and inhomogeneity of the surface. When the value of the parameter *n* is closer to 1, it means that the surface is more homogeneous and smooth [48,58,59].

The corresponding transfer function of the total impedance for the equivalent circuit is given by the following equation [60]:

(8) $$Z_{total}=R_{s}+{\left(Q\cdot {\left(j\omega \right)}^{n}+{\left(R_{1}+W\right)}^{-1}\right)}^{-1}$$

The values of capacitance (*C*_1_) were calculated according to the following equation [21,61]:

(9) $$C_{1}=\frac{{\left(R_{1}\cdot Q\right)}^{\frac{1}{n}}}{R_{1}}$$

**Figure 9:**
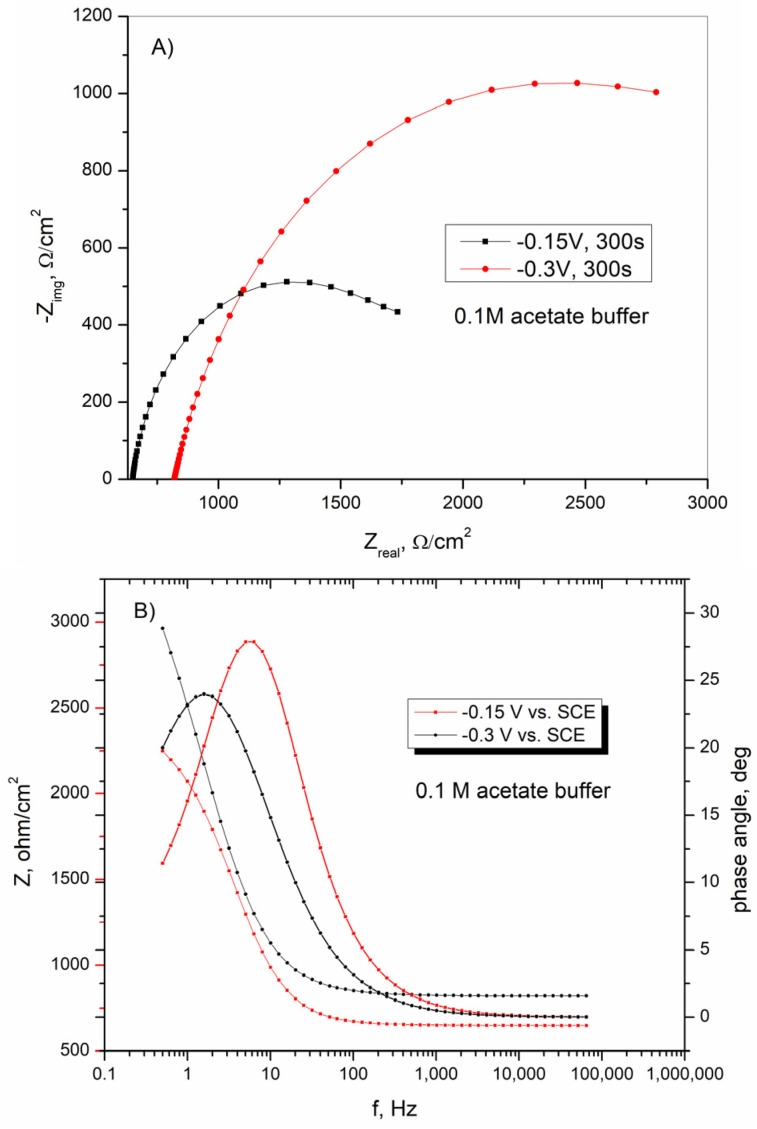
(**A**) Nyquist plot and (**B**) Bode diagrams of BiFE obtained in acetate buffer solution after Bi(III) deposition on Cu37Zn substrate by chronoamperometry at different potentials.

According to the Nyquist diagram shown in Figure 9A, the lower impedance loop indicates a lower polarization resistance value associated with higher electrode sensitivity. The curves shown in Figure 9A indicate that BiFE formed at a potential of −0.15 V vs. SCE, which has higher sensitivity than BiFE and forms at a potential of −0.3 V vs. SCE [57,61]. According to the results, the kinetically-controlled process is followed by the diffusion controlled process represented by the Warburg element. The diffusion-controlled process decreases the electron transfer rate and affects the overall response, ultimately leading to lower electrode sensitivity [57]. The values of W shown in the Table 2 are higher for BiFE formed at −0.3 V vs. SCE, indicating the lower sensitivity of the bismuth film electrode formed at this potential. According to the results presented in Table 2, the Q value is lower when the bismuth film was formed at a potential of −0.15 V vs. SCE, indicating better adsorption of Bi(III) on the electrode surface. The Bode plots of BiFE obtained in acetate buffer solution at different potentials are shown in Figure 9B. The impedance behavior of BiFE is the result of the morphological structure of the deposits formed in addition to the electrode material and the solution used. In these experiments, the electrode material and the solution used were the same, so the observed impedance behavior of the bismuth film electrode is only the result of the morphological structure [62]. The curves shown in Figure 9B show that the BiFE had a lower impedance when the bismuth film was formed at a potential of −0.15 V vs. SCE. According to the Bode phase angle plots (Figure 9B), the impedance in the high-frequency range is independent of the frequency change, and the phase angle approaches 0°, indicating a resistive behavior. In the mid-frequency range, capacitive behavior is observed while, in the low frequency range, the phase angle reaches lower values for BiFE, which was formed at a potential of −0.3 V vs. SCE, indicating a more resistive behavior. The surface of the BiFE obtained at this potential is rougher and provides more places for water molecules to penetrate, resulting in a more resistive electrode [63].

The quantitative measure of surface roughness is calculated using the following equation [64]:

(10) $$d_{s}=3-n$$

where ds is the fractal dimension of the surface and n is the CPE parameter.

The calculated values of the fractal dimension of the surface range from 2 to 3. When d_s_ → 2, the electrode surface is flat, and, when d_s_ → 3, the surface is rough [64]. The calculated values are listed in the Table 2 and indicate that the BiFE surface was rougher when the film was formed at a potential of −0.3 V vs. SCE.

**Figure 10:**
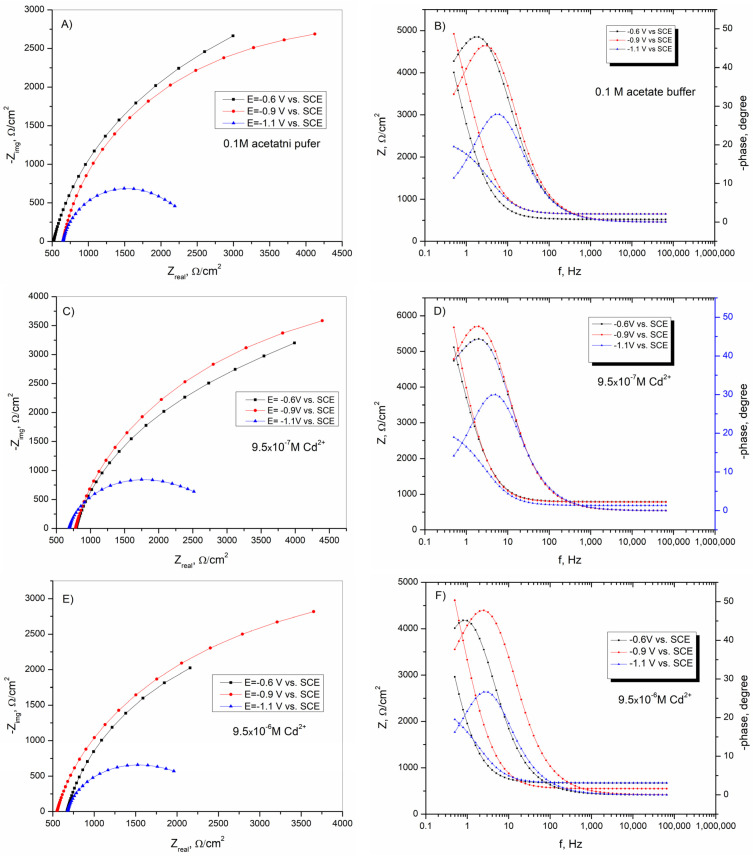
The EIS measurements (dots) and the fitted response (lines) for the BiFEs in acetate buffer solution with and without the addition of different amounts of Cd^2+^; (**A**,**C**,**E**) Nyquist plot; (**B**,**D**,**F**) Bode diagrams.

In Figure 10, EIS spectra of BiFEs in acetate buffer solution, with and without the addition of different concentrations of cadmium ions at three different potentials, are shown to determine the characteristics of the formed electrodes before and after the stripping signal. In the Bode diagrams (Figure 10B,D,F), three regions can be seen at low, medium, and high frequencies. At the high frequencies, the phase angle has a value of 0°. The values of log |Z| are independent of the change of log f and remain unchanged, which is characteristic of a resistor. In the middle range, the phase angle increases at more positive potentials (Figure 10B,D,F). In the same range, a linear correlation between log |Z| and log f was also observed, which is typical of a capacitor. In the low-frequency range, the phase angle dependence of frequency and the log |Z| dependence of log f indicate the total resistance of the system, represented by the polarization resistance and the Warburg diffusion element. The spectra of the Bode diagrams in the low-frequency range indicate a stronger diffusion-controlled process, which was more pronounced at more positive potentials.

**Figure 11:**
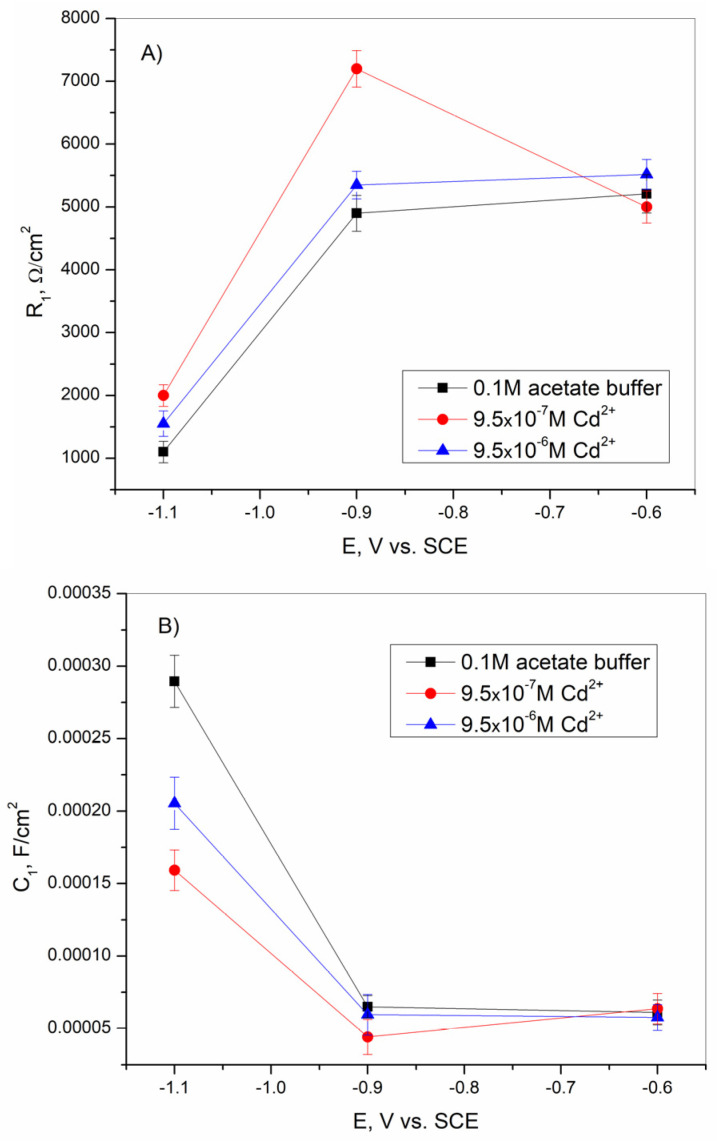
The variation in (**A**) R_1_ and (**B**) C_1_, at different potentials, as calculated during the fitting procedure for BiFE with and without the addition of different amounts of Cd^2+^. The mean values of three measurements are presented with corresponding error bars.

Figure 11 shows the change in the values of R_1_ and C_1_ for the BiFEs with and without the presence of different concentrations of Cd^2+^ at different potentials. According to the results presented, the R_1_ values increased sharply with increasing potential from −1.1 V to −0.9 V vs. SCE. Then, the R_1_ values increased slightly at a potential of −0.6 V vs. SCE in the bare acetate buffer solution and in solution with the addition of cadmium at a concentration of 9.5–10^−6^ M. In solution with an amount of 9.5–10^−7^ M Cd^2+^, R_1_ decreased at a potential of −0.6 V vs. SCE. On the other hand, C_1_ values decreased sharply at a potential of −0.9 V vs. SCE compared to C_1_ values at −1.1 V vs. SCE. A slight increase in C_1_ is observed at the potential shift from −0.9 to −0.6 V vs. SCE. The addition of cadmium ions affected the R_1_ and C_1_ shifts at −1.1 and −0.9 V vs. SCE, respectively. At these potentials, R_1_ increased, and C_1_ decreased. At a potential of −0.6 V vs. SCE, the presence of Cd^2+^ did not significantly affect the shift in R_1_ and C_1_ values.

According to the results shown in Figure 11, the higher value of R_1_ in the presence of the lower concentration of Cd^2+^ ions indicates the deposition of a smaller amount of cadmium on the BiFE surface, resulting in a lower sensitivity of the method [65]. In the observed potential range of from −1.1 to −0.6 V vs. SCE, the potential of zero charge (PZC) was defined as the lowest C_1_ value and was reached at a potential of −0.9 V vs. SCE [21,61]. The potential of zero charge is an important parameter in adsorptive stripping voltammetry since adsorption processes usually occur at this potential [65].

#### 3.3.3. Optimization of Parameters for Determination of Cd^2+^

**Figure 12:**
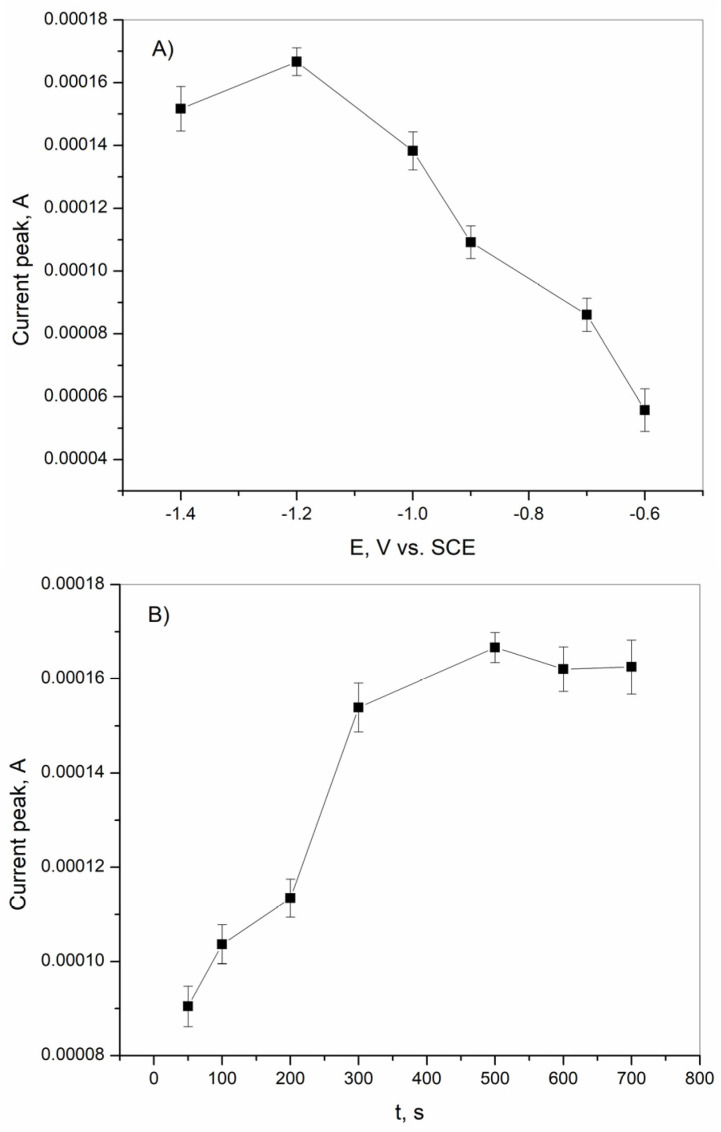
(**A**) Effect of deposition potential on the Cd^2+^ determination in acetate buffer solution at the BiFE. (**B**) Effect of deposition time on the Cd^2+^ determination in acetate buffer solution at the BiFE. The concentration of cadmium was 1.33 × 10^−5^ M. The mean values of three measurements are presented with corresponding error bars.

The optimal deposition potential for the maximum analytical signal of cadmium was investigated in a wide potential range from −1.4 V vs. SCE to −0.6 V vs. SCE, and the results are shown in Figure 12A. In addition to the effect of deposition potential, the effect of deposition time on the stripping peak of Cd^2+^ was also investigated. The results of this investigation are shown in Figure 12B. The effect of the deposition potential on the stripping signal varies in the studied range. In the range from −0.6 V vs. SCE to −1.2 V vs. SCE, the analytical signal increases and reaches a maximum at a deposition potential of −1.2 V vs. SCE. A further negative shift of the deposition potential leads to a decrease in the stripping peak due to the evolution of the hydrogen and the decrease in the number of active sites on the BiFE surface [66,67]. Accordingly, a potential of −1.2 V vs. SCE was chosen as the optimal deposition potential for further experiments. The deposition time, which was investigated in the range of from 50 s to 700 s, shows a high influence on the stripping signal. Two trends of the peak current behavior can be observed on the obtained curve. The first extends over a range from 50 s to 500 s, where the analytical signal increases due to the enhanced reduction of cadmium ions. The second one starts from 500 s to 700 s and is characterized by the decrease in the stripping peak as a result of the saturation of the active sites on the bismuth film electrode [66].

**Figure 13:**
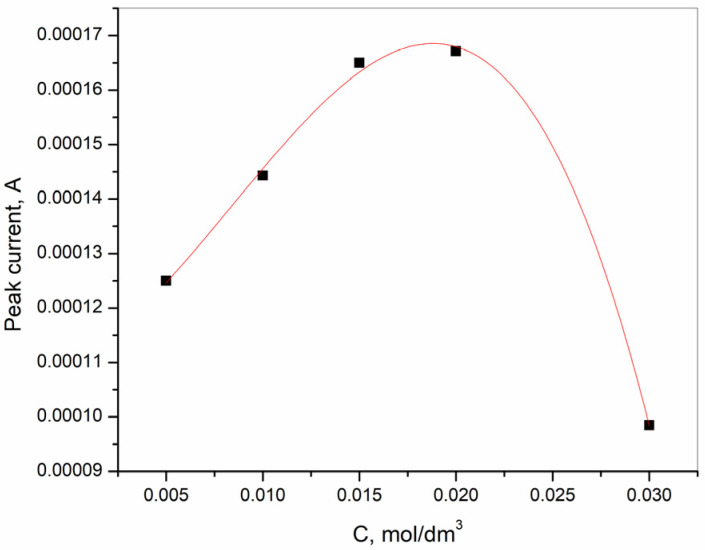
Effect of the Bi(III) concentration on the analytical signal on the Cd^2+^ determination in acetate buffer solution.

Figure 13 shows the effects of Bi(III) concentration in the range of from 0.005 to 0.03 M on the analytical signal obtained in the acetate buffer solution. The results show that, in the concentration range of from 0.005 to 0.02 M, the stripping signal increases as a result of the formation of an alloy between Bi(III) and cadmium, which enhances the enrichment of the target metal. At a Bi^3+^ concentration of 0.03 M, the current peak decreases rapidly due to the low transfer of metal ions, which is due to the formation of a thick film. The dependence of the analytical signal on the thickness of the bismuth film was found in several studies [66,68,69]. The optimum concentration for the preparation of BiFE for the purpose of determining Cd^2+^ is, therefore, 0.02 M Bi^3+^, which was used in further experiments.

### 3.4. The Determination of Cd^2+^ Ions

**Figure 14:**
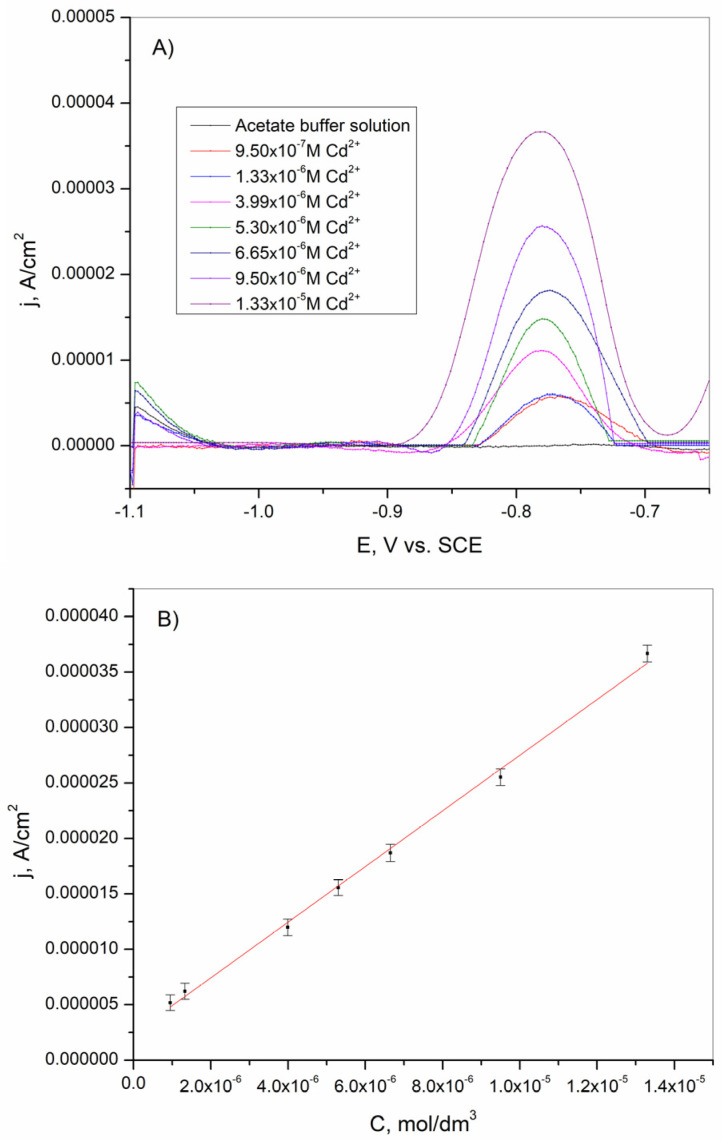
(**A**) Square-wave anodic stripping voltammogram of Cd^2+^ from 9.5 × 10^−7^ to 1.33 × 10^−5^ M in 0.1 M acetate buffer solution. (**B**) Calibration curve for Cd^2+^_._ The mean values of three measurements are presented with corresponding error bars.

The square-wave stripping voltammetry (SWV) technique is recognized as one of the most sensitive analytical methods and was used to evaluate the limit of detection (LOD) and limit of quantification (LOQ). The concentration of Cd^2+^ ions in the tested solution was in the range from 9.5 × 10^−7^ to 1.33 × 10^−5^ M in the acetate buffer solution. The obtained results presented in Figure 14A indicate that an increase in the Cd^2+^ concentration leads to an increase in the peak current. Also, from Figure 14A, it is evident that the stripping peak for cadmium is well-defined. The calibration curve for cadmium determination was obtained from results acquired by the square-wave stripping voltammetry. The calibration curve is shown in Figure 14B. The linear relationship that was achieved in the examined concentration range can be described by the following equation:

(11) $$I_{p}\left(A\right)=2.3869\times 10^{-6}+2.5145C_{{Cd}^{2+}}\left(M\right)\mathrm{with}\;R^{2}\;=\;0.997$$

The limit of detection and limit of quantification were determined according to the following equations:

(12) $$LOD=\frac{3S}{b}$$

(13) $$LOQ=\frac{10S}{b}$$

where *S* represents the standard deviation of the peak currents, while b is the slope of the calibration curve. The obtained values for LOD and LOQ were 5.045 × 10^−7^ M and 1.685 × 10^−6^ M, respectively. The limit of quantification is the lowest amount of heavy metal that may be detected and included as an acceptable result. The limit of detection is the lowest amount of heavy metal that can be detected in tested solutions. The linear range and limit of detection of Cd^2+^ obtained by various BiFEs are presented in Table 3.

The repeatability and reproducibility of the synthesized BIFEs were investigated in the presence of 3.99 × 10^−6^ M Cd^2+^. Four electrodes were prepared, and SWV measurements were performed under optimal conditions to investigate the reproducibility of the BIFEs. The relative standard deviations (RSDs) were calculated, and the obtained value of 3.86% indicates a good reproducibility of the tested sensor.

The repeatability was investigated in the presence of 1.33 × 10^−5^ M Cd^2+^. The relative standard deviation was calculated after five measurements, and the obtained value of 4.59% indicates a good repeatability of the synthesized BIFE in the tested environment.

It can be concluded, based on the data presented in Table 3, that most of the Bi-film modifications were performed on carbon-based electrodes, such as GCE, PGE, etc. However, the ease of preparation and shaping, as well as good LOD and LOQ for Cd^2+^ detection, can be considered the main advantages of metal-substrated electrodes. To the best of our knowledge, there are no previous results on the modification of brass electrodes with Bi-film, whereas the complex hierarchical structures obtained by Bi electrodeposition on S-doped Cu/brass mesh (Bi/Cu-S/BM) were tested as electrode materials for the electrocatalytic reduction of CO_2_ [82]. Additionally, brass was used for glucose sensors where selective and precise oxidation enabled the formation of a composite oxide surface [83]; it was also included in the testing of a potential electronic tongue for vinegar and fruit juices [84].

### 3.5. Interference Investigation

**Figure 15:**
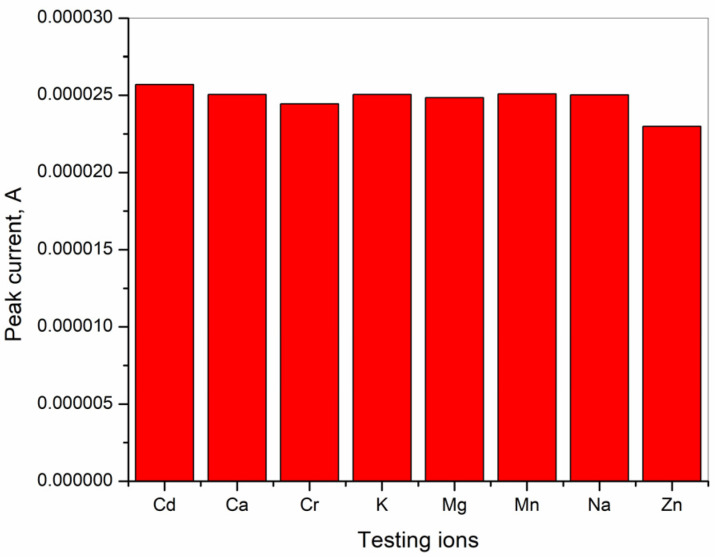
Selectivity of bismuth film electrode for detection of Cd^2+^.

An interference study of different cations for the determination of cadmium was performed. Selectivity analysis was accomplished in the presence of a 10-fold higher concentration of the following ions: Cr^3+^, Mn^2+^, and Zn^2+^, and with the addition of 100-fold exceeded concentrations of Ca^2+^, K^+^, Mg^2+^, and Na^+^ in the presence of 9.5 × 10^−6^ M Cd^2+^. According to the results presented in Figure 15, there are no detected changes in the stripping signal, which reveals that the tested cations do not have an influence on the determination of the Cd^2+^ ions in the tested solution. This proves the good selectivity of the tested BiFE electrode, which provides a good possibility for the use of the electrode in real samples.

### 3.6. Real Sample Analysis

**Figure 16:**
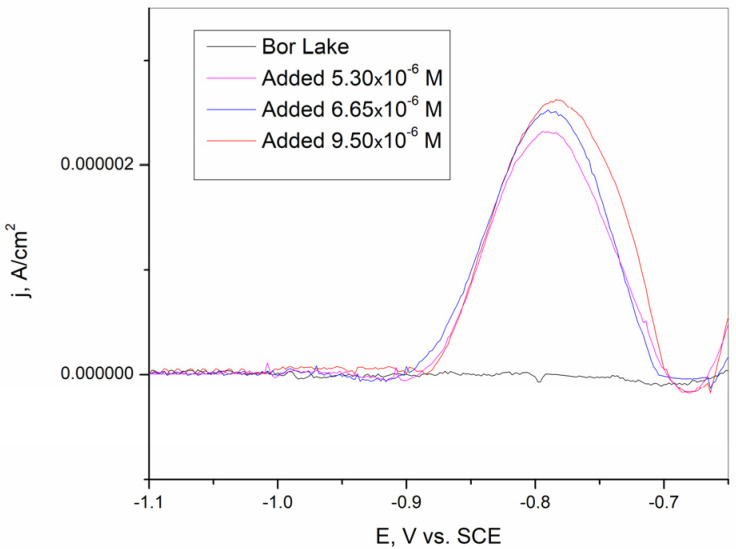
Square-wave stripping voltammogram of Cd^2+^ in spiked Bor Lake samples on synthesized BiFE on the brass substrate.

The synthesized BiFE was tested for the determination of Cd^2+^ in Bor Lake samples. Since Cd^2+^ could not be detected in the real Bor Lake samples, 5.3 × 10^−6^, 6.65 × 10^−6^, and 9.5 × 10^−6^ M Cd^2+^ was added to the samples before testing (BL 1, BL 2, and BL 3). After the samples were prepared, square-wave stripping voltammetry measurements were performed, and the results are shown in Figure 16 and Table 4. The prepared BiFE shows a good recovery for Bor Lake samples in the range of from 99.4 to 100.7% and a relative standard deviation (RSD) in the range of from 1.86 to 3.57. The results obtained show that the BiFE prepared on a brass substrate provides satisfactory results for the determination of Cd^2+^ in the real samples investigated.

## 4. Conclusions

This paper presents the results of the production of a bismuth film electrode on a brass surface for use as a sensor and describes its application for the determination of cadmium. Based on the chronoamperometric curves, it was found that a thicker film was formed on the brass surface with an increasing bismuth concentration. The EIS measurements show that a potential of −0.15 V vs. SCE is more suitable for the deposition of Bi(III) on the brass surface. The BiFE produced at this potential has a higher sensitivity, which ensures a stronger stripping signal. The EIS measurements were performed at potentials more negative than the potential at which the stripping signal was observed and at a potential of −0.6 V vs. SCE, which is slightly more positive than the potential at which the Cd^2+^ peak occurs. The EIS measurements at these potentials aim to determine the behavior of the BiFE near the potential at which the current peak was reached, in particular, the behavior of the electrode before the stripping signal. The values obtained for R_1_ and C_1_ at different potentials indicate that a negative potential causes better deposition of cadmium ions and a higher sensitivity of the electrode. The EIS studies show that the potential of zero charge was achieved at a potential of −0.9 V vs. SCE, which is recognized as the potential at which the adsorption process occurs. The EIS results also show that the presence of cadmium at the concentrations tested had no significant effect on the resistive and capacitive behavior of the electrode.

According to the results presented in this study, the SWV method with the use of a bismuth film electrode on a brass surface can ensure a sensitive and reliable method for the determination of cadmium in acetate buffer solutions. In the concentration range investigated (9.5 × 10^−7^–1.33 × 10^−9^ M), the calibration curve achieved a satisfactory linearity with very good repeatability (RSD 4.59%) and reproducibility (RSD 3.86%). The LOD value was found to be 5.045 × 10^−7^ M, and the LOQ value was 1.685 × 10^−6^ M, which is comparable to the literature data. In addition, BiFE was observed to be effective in the determination of Cd^2+^, even at interferences 10-fold (Cr^3+^, Mn^2+^, Zn^2+^) and 100-fold (Ca^2+^, K^+^, Mg^2+^, Na^+^) higher than the measured value, indicating that the investigated bismuth film electrode has potential for use in real systems. The analysis of samples from the Bor Lake shows that BiFE prepared on a brass substrate can be used for the determination of Cd^2+^ in real samples.

## Figures and Tables

**Table 1:** *AN*_∞_ and *N_s_* of Bi(III) on the brass electrode.

*E*, V vs. SCE	*AN*_∞_, cm^−2^ s	*N_s_*, cm^−2^
−0.15	3.65 × 10^6^	1.27 × 10^6^
−0.2	1.08 × 10^5^	1.13 × 10^5^
−0.3	3.59 × 10^4^	5.41 × 10^4^

**Table 2:** Electrochemical impedance spectroscopy parameters for BiFE in acetate buffer solution.

Bi(III) Deposition Potential, V vs. SCE	*R_S_*, Ωcm^2^	*R*_1_, Ωcm^2^	*W*, Ω^−1^cm^−2^s^0.5^	*Q*, Ω^−1^s^n^	*n*	*d_s_*
−0.15	6.48 × 10^2^	1.65 × 10^3^	2.05 × 10^2^	1.02 × 10^−5^	0.86	2.14
−0.3	8.2 × 10^2^	2.5 × 10^3^	5.75 × 10^2^	6.35 × 10^−5^	0.8	2.2

**Table 3:** Linear range and limit of detection of Cd^2+^ of bismuth and Bi-film-modified electrodes.

Electrode	Modifier	Method	Linear Range	LOD	Ref.
Battery graphite electrode	/	ASV	5–1000 ppb	115.37 ppb	[70]
	Bi, MWCNT, Nafion			1.06 ppb	
PGE	Bi nanoparticle/Nafion	ASV	10–150 μg/L	7.31 μg/L	[71]
Graphite-polyurethane composite electrodes	Bismuth	SWASV		2.2 nmol/L	[72]
Pencil-lead graphite	Bismuth	DPASV	48.3–233 μg/L	11.0 μg/L	[34]
GCE	Bismuth	ASV	10–200 μg/L	0.11 μg/L	[73]
GCE	/	SWASV	10.0–357.1 μg/L	3.2 μg/L	[65]
	0.5 ppm Bi(III)		5.4–72.8 μg/L	0.7 μg/L	
	1.0 ppm Bi(III)			0.3 μg/L	
GCE	Bismuth	DPASV	5.0–110.0 ppb	0.93 ppb	[74]
GCE	NanoSiO_2_–CTS/BiFE	DPASV	0–40 μg/L	0.1 μg/L	[75]
GCE	1.0Bi0.0Sn_1.0_mg/L	SWASV	~2–225 μg/L	0.3 μg/L	[57]
	0.8Bi0.2Sn_0.5_mg/L and 0.6Bi0.4Sn_1.0_mg/L			0.5 μg/L	
GCE	1.0Bi0.0Sb_1.0_mg/L	SWASV		0.3 μg/L	[76]
	0.1Bi0.9Sb_1.0_mg/L		2.2–425.1 μg/L	1.2 μg/L	
GCE	0.8Bi0.2Cu/1.0 mg/L	SWASV	~380–1120 μg/L	9.2 μg/L	[77]
GCE	Bismuth B6_prod_ B6_sum_	SWASV	10.7–54.9 μg/L	0.4 μg/L	[78]
			3.8–13.8 μg/L		
GCE	Bi-graphdiyne	DPASV	2.0–100 μM	0.367 μM	[79]
CNTs/SGC	Bismuth	ASV	2 × 10^−9^–2 × 10^−7^ M	6.2 × 10^−10^ M	[80]
CPEs	Bismuth	SWASV		0.15 μg/L	[81]
Bi bulk electrode	/	ASV	10–100 μg/L	54 ng/L	[27]
Cu	Cu/Nafion/Bi	DPV	2–12 μg/L	0.68 μg/L	[35]
Brass	Bismuth	SWV	9.5 × 10^−7^–1.33 × 10^−5^ M	5.045 × 10^−7^ M	this work

Anodic stripping voltammetry (ASV); square-wave stripping voltammetry (SWV); differential pulse anodic stripping voltammetry (DPASV); square-wave anodic stripping voltammetry (SWASV); carbon nanotubes/spherical glassy carbon powder (CNTs/SGC).

**Table 4:** Determination of Cd^2+^ in spiked Bor Lake samples using synthesized BiFE (N = 3).

Sample	Added Cd^2+^, M	Found Cd^2+^, M	Recovery, %	RSD
BL 1	5.3 × 10^−6^	(5.34 ± 0.19) × 10^−6^	100.7	3.57
BL 2	6.65 × 10^−6^	(6.66 ± 0.13) × 10^−6^	100.2	1.95
BL 3	9.5 × 10^−6^	(9.44 ± 0.18) × 10^−6^	99.4	1.86

## Data Availability

Dataset available on request from the authors. The raw data supporting the conclusions of this article will be made available by the authors on request.
